# Two new species of *Entoloma* subgenus *Cyanula* (*E. alpigenum* and *E. muscicola*) from montane forests of Azad Jammu and Kashmir, Pakistan (Agaricales, Entolomataceae)

**DOI:** 10.3897/mycokeys.130.182629

**Published:** 2026-03-18

**Authors:** Rubab Khurshid, Arooj Naseer, Qirui Li, Abdul Nasir Khalid

**Affiliations:** 1 State Key Laboratory of Discovery and Utilization of Functional Components in Traditional Chinese Medicine & School of Pharmaceutical Sciences, Guizhou Medical University, Guian New District, Guiyang, Guizhou 550004, China Institute of Botany, University of the Punjab Lahore Pakistan; 2 Fungal Biology and Systematics Laboratory, Institute of Botany, University of the Punjab, Quaid-e-Azam Campus, 54590, Lahore, Pakistan State Key Laboratory of Discovery and Utilization of Functional Components in Traditional Chinese Medicine & School of Pharmaceutical Sciences, Guizhou Medical University Guiyang China

**Keywords:** Entolomataceae, mixed and coniferous forests, multigene phylogeny, mushrooms, new species

## Abstract

Two new species of genus *Entoloma* in subgenus *Cyanula*, *Entoloma alpigenum* and *Entoloma muscicola* are described from the mixed forests of Azad Jammu and Kashmir, Pakistan. *Entoloma muscicola* is characterized by a cuspidate, deep brown pileus with wavy margins, distant pale yellow lamellae, weakly angled mono-gutulate basidiospores, predominantly two-spored basidia, and clavate cheilocystidia with wavy apical caps. In contrast, *Entoloma alpigenum* is characterized by a light brown, slightly squamulose pileus, pale yellowish lamellae, larger 5–7 angled mono-gutulate basidiospores and cylindrical to flexuose cheilocystidia. Both species are ecologically associated with high-altitude mixed forests. Phylogenetic analyses have placed these new species in subgenus *Cyanula*, forming sister clades within section *Griseocyanea*, which support their distinct taxonomic status. Illustrated descriptions of their macro- and microscopic features and phylogenetic analyses, based on nrITS, nrLSU, mtSSU and *rpb*2 regions are provided.

## Introduction

*Entoloma* (Fr.) P.Kumm. (Agaricales, Entolomataceae) is one of the largest and most morphologically diverse genera of agaricoid fungi. It currently comprises about 2,000 species distributed worldwide from tropical to temperate regions (Noordeloos et al. 2022a). These species are characterized by pinkish to reddish-brown spore prints, angular basidiospores with distinct facets, and typically adnate lamellae (Co-David et al. 2009). They exhibit remarkable morphological diversity, ranging from mycenoid and collybioid to tricholomatoid and omphalinoid forms, making taxonomic delimitation challenging (Co-David et al. 2009; Noordeloos and Gates 2012; Morgado et al. 2013; He et al. 2019).

Most species of *Entoloma* are terrestrial saprotrophs involved in litter decomposition, though some are biotrophic or ectomycorrhizal (Co-David et al. 2009; Morgado et al. 2013). Historically, *Cyanula* was introduced by Romagnesi (1974) as a section within *Rhodophyllus* Quél. (= *Entoloma*) based on morphological characters. The combination *Entoloma* sect. *Cyanula* (Romagn.) Noordel. was later included within *Entoloma* subg. *Leptoniasensu lato*. Traditionally, *Leptonia* was divided into three sections *Leptonia*, *Cyanula*, and *Griseorubida* (Noordeloos 2004) but subsequent molecular analyses revealed this grouping to be polyphyletic. Section *Leptonia* belongs to the *Nolanea*–*Claudopus* clade, while *Cyanula* and *Griseorubida* cluster within the *Inocephalus*–*Cyanula* clade (Co-David et al. 2009). Morphologically, species of sect. *Leptonia* exhibit clamp connections, whereas those of *Cyanula* lack them. Based on these distinctions, sect. *Cyanula* was elevated to subgenus rank (Noordeloos and Gates 2012; Noordeloos et al. 2022a).

Members of subg. *Cyanula* are typically recognized by their bluish to violaceous basidiomata, smooth basidiospores, absence of clamp connections, and ecological preference for woodland habitats. *Entoloma* species have been well studied in Europe and East Asia, their diversity in South Asia remains poorly documented. In Pakistan, only 14 taxa of *Entoloma* have been reported (Ahmad et al. 1997; Izhar 2022; Fatima et al. 2023; Khalid et al. 2024; Khan et al. 2026), and molecular data remain scarce. During a survey of mycobiota in Azad Jammu and Kashmir, Pakistan, two new species of *Entoloma* subgenus *Cyanula* were discovered. These new species are described based on morphology and multigene phylogenetic analyses based on four markers nrITS, nrLSU, mtSSU and *rpb*2.

## Material and methods

### Morphological and microscopic studies

Basidiomata were collected from coniferous mixed forests comprising *Pinus*, *Cedrus*, and *Abies* species in District Bagh, Azad Jammu and Kashmir, during the rainy seasons of 2019–2021. In situ, photographs of the basidiocarps were taken using Nikon D70S and D300S camera. Morphological characteristics were recorded from fresh specimens, and colors were designated using the mColorMeter application (Yanmei He, Mac App Store). Voucher specimens of the newly described species were deposited in the Herbarium of the Institute of Botany, University of the Punjab, Lahore, Pakistan.

Microscopic examination was performed on freehand sections of fresh and dried specimens mounted in 5% (w/v) aqueous potassium hydroxide (KOH) solution, using a Meiji Techno MX4300H compound microscope. A total of 30 basidiospores, basidia, cystidia, and hyphae from the pilei were measured from each collection. The notation “n/m/p” denotes the number of basidiospores measured from m fruit bodies of p collections. Basidiospore dimensions are presented as length × width (L × W), average length × average width (av. L × av. W), and average quotient (av. Q), with extreme values given in parentheses. The range represents at least 90% of the values. Measurements include the arithmetic mean of spore length and width. Additionally, scanning electron microphotographs were obtained at the University of Vienna, Austria.

### Molecular study

#### Extraction, amplification and sequencing of DNA

Genomic DNA was extracted using either the DNeasy Plant Mini Kit (QIAgen GmbH, Hilden, Germany) or the Thermo Scientific GeneJET Plant Genomic DNA Purification Kit (Thermo Fisher Scientific Inc., Waltham, Mass., USA). Four regions were amplified and sequenced: ITS, LSU, mtSSU, and *rpb*2. The primer pairs used were ITS4/ITS5 (White et al. 1990) for ITS; LR0R/LR5 (Vilgalys and Hester 1990) for LSU; brpb2-6F/brpb2-7.1R or frpb2-7cR (Matheny 2005) for *rpb*2 and MS1 & MS2 (White et al. 1990) for mtSSU. PCR was performed in a 10 μL reaction volume consisting of 5 μL Biomed 2× Taq Plus PCR MasterMix (Biomed, Beijing, China), 3.9 μL ddH_2_O, 0.3 μL of each primer (10 μmol/L), and 0.5 μL DNA template. PCR products were purified using an enzymatic PCR cleanup (Werle et al. 1994) as described by Voglmayr and Jaklitsch (2008). DNA was cycle-sequenced using the ABI PRISM Big Dye Terminator Cycle Sequencing Ready Reaction Kit v3.1 (Applied Biosystems, Warrington, UK). Sequencing was performed on an automated DNA sequencer (ABI 3730xl Genetic Analyzer, Applied Biosystems). Chromatograms were checked and assembled using SeqmanII v.5.07 (Dnastar Inc.).

#### Phylogenetic analyses

A total of 149 sequences (42 ITS, 33 LSU, 15 *rpb*2, 17 mtSSU given in Table 1) were used to construct multigene phylogram based on Bayesian inference (BI) and Maximum likelihood (ML). The selection of sequences for the phylogenetic analyses were based on the results of ITS BLAST and from recent articles (Noordeloos et al. 2022b; Chen et al. 2025; Khan et al. 2026). Three species of *Entoloma* subg. *Nolanea* i.e *E. sericeum* and *E. atropapillatum* were designated as outgroups. Sequences for each locus were aligned separately using MAFFT (online server) with automatic algorithm selection (Katoh et al. 2019). First, phylogenetic trees were constructed separately for each region and their congruence were checked. Bayesian inference (BI) was conducted in MrBayes v.3.2.7a (Ronquist et al. 2012) with each partition (ITS, LSU, RPB2, mtSSU) assigned a GTR+I+G substitution model. Maximum likelihood (ML) analysis in IQ-TREE v.2.1.2 (Nguyen et al. 2015), was performed using the same GTR+I+G model for all partitions, with 1,000 ultrafast bootstrap replicates to assess node support.

**Table 1.** Sequences used for phylogenetic analyses in this study. Newly generated sequences are shown in and ‘–’ indicating sequence unavailability.

Species	Voucher/strain	Country	GenBank Accession Numbers				Reference
			ITS	LSU	*rpb2*	mtSSU	
***Entoloma alpigenum***	**LAH38754**	**Pakistan**	**PX687957**	**PX687958**	**PX694301**	**PX693682**	**This study**
***E. alpigenum***	**WU48890**	**Pakistan**	**PX696031**	**PX687959**	**PX694300**	**–**	**This study**
***E. alpigenum***	**NALA-030**	**Pakistan**	**PX696032**	**PX687960**	**–**	**PX693683**	**This study**
*Entoloma ammophilum*	L0608224	Netherlands	MW934591	MZ333146	–	–	Crous et al. (2021a)
*E. atropapillatum*	FK0898	Brazil	KF679354	KF738940	MH190107	KF738929	Karstedt et al. (2020)
*E. brunneicoeruleum*	L0608198	Brazil	MZ145166	–	–	–	Dima et al. 2021
*E. caespitosum*	GDGM24025	China	JQ281490	JQ410327	OR916407	OR912420	He et al. (2012)
*E. caespitosum*	GDGM27564	China	JQ281477	JQ320130	JQ993078	JQ993070	He et al. (2012)
*E. fuscosquamosum*	iNAT:30847414	USA	MZ268031	–	–	–	Unpublished in GenBank
*E. griseocyaneum*	LE254351	Russia	KC898444	KC898498	–	KC898463	Morozova et al. (2014)
*E. griseocaeruleum*	CME13	Panama	MZ611624	MZ611624	–	–	Reschke et al. (2022a)
*E. holmvassdalenense*	O:F-304575	Norway	MZ869018	MZ678746	–	–	Reschke et al. (2022a)
*E. incanum*	LE311794	Russia	OK161249	OK161276	–	–	Crous et al. (2021b)
*E. incanum*	LE312503	Sweden	OK161247	OK161275	–	–	Crous et al. (2021b)
*E. mastoideum*	D321	Thailand	OR066303	OR066336	–	OR066375	Pringsulaka et al. (2024)
*E. mastoideum*	D322	Thailand	OR066304	OR066337	–	OR066376	Pringsulaka et al. (2024)
*E. montanum*	GB:0191631	Sweden	MW340897	–	–	–	Noordeloos et al. (2021)
*E. montanum*	OF-252062	Norway	MW340877	–	–	–	Noordeloos et al. (2021)
***E. muscicola***	**LAH38755**	**Pakistan**	**PX687953**	**PX687956**	**PX693684**	**PX693681**	**This study**
***E. muscicola***	**WU48891**	**Pakistan**	**PX687954**	**PX687955**	**PX694299**	**PX693680**	**This study**
*E. nigroflavescens*	FA1726	France	MZ198883	–	–	–	Buyck et al. (2022)
*E. nigroflavescens*	FA4277	France	MZ198884	–	–	–	Buyck et al. (2022)
*E. orientosinense*	HFJAU2920	China	PQ584688	PQ584708	PQ617187	–	Chen et al. (2025)
*E. orientosinense*	HFJAU4048	China	PQ584689	PQ584709	PQ617188	–	Chen et al. (2025)
*E. praegracile*	GDGM29251	China	JQ281482	JQ320129	JQ993077	JQ993072	He et al. (2013)
*E. serrulatum*	LE254361	Russia	KC898447	KC898501	–	KC898466	Morozova et al. (2014)
*E. sericeum*	KaiR1259	Sweden	OL338182	OL338182	OL405219	OL338493	Reschke et al. (2022b)
*E. sericeum*	KaiR237	Germany	OL338118	OL338542	OL405220	OL338494	Reschke et al. (2022b)
*E. subfarinaceum*	127466550	USA	OP470463	–	–	–	Unpublished in GenBank
*E. subfarinaceum*	SAT1518702	USA	KY777374	–	–	–	Noordeloos et al. (2021)
*E. subpraegracile*	HFJAU3878	China	PQ584693	PQ584720	PQ617199	–	Chen et al. (2025)
*E. subpraegracile*	HFJAU5175	China	PQ584703	PQ584715	PQ617192	–	Chen et al. (2025)
*E. subpraegracile*	HFJAU5115	China	PQ584699	PQ584713	PQ617194	–	Chen et al. (2025)
*E. subpraegracile*	HFJAU5140	China	PQ584702	PQ584714	PQ617195	–	Chen et al. (2025)
*E. subtenuicystidiatum*	GDGM29246	China	JQ320114	JQ320132	–	–	He et al. (2013)
*E. subtenuicystidiatum*	GDGM28459	China	JQ320109	JQ320116	–	JQ99307	He et al. (2013)
*E. turci*	FA4328	France	OR419871	–	–	–	Unpublished in GenBank
*E. turci*	F-2783	Sweden	PQ652635	PQ652635	–	–	Unpublished in GenBank
*E. violaceoserrulatum*	JV8329F(TUR)	Finland	MF476913	MF487803	–	MF476923	Morozova et al. (2018)
*E. wuyishanense*	HFJAU3871	China	PQ584692	PQ584717	PQ617197	–	Chen et al. (2025)
*E. wuyishanense*	HFJAU3874	China	PQ584694	PQ584718	PQ617198	–	Chen et al. (2025)
*E. yanacolor*	QCAM6312	Ecuador	MG947210	MG947210	–	–	Crous et al. (2018)

## Results

### Molecular phylogenetic analyses (Fig. 1)

The combined dataset included 3780 nucleotide sites (ITS: 1–808 bp; LSU: 809–1910 bp; *rpb*2: 1911–3179 bp and mtSSU: 3179–3780 bp) including gaps. The bestfit evolutionary model selected for the concatenated analysis was TIM2+F+I+G4 model. The BI phylogenetic analysis was stopped after 5,400,000 generations, when the average standard deviation of split frequencies converged to 0.009874. For the maximum likelihood (ML) analysis, models were selected using the Bayesian Information Criterion (BIC), yielded a final log-likelihood value of -18410.138.

**Figure 1.**
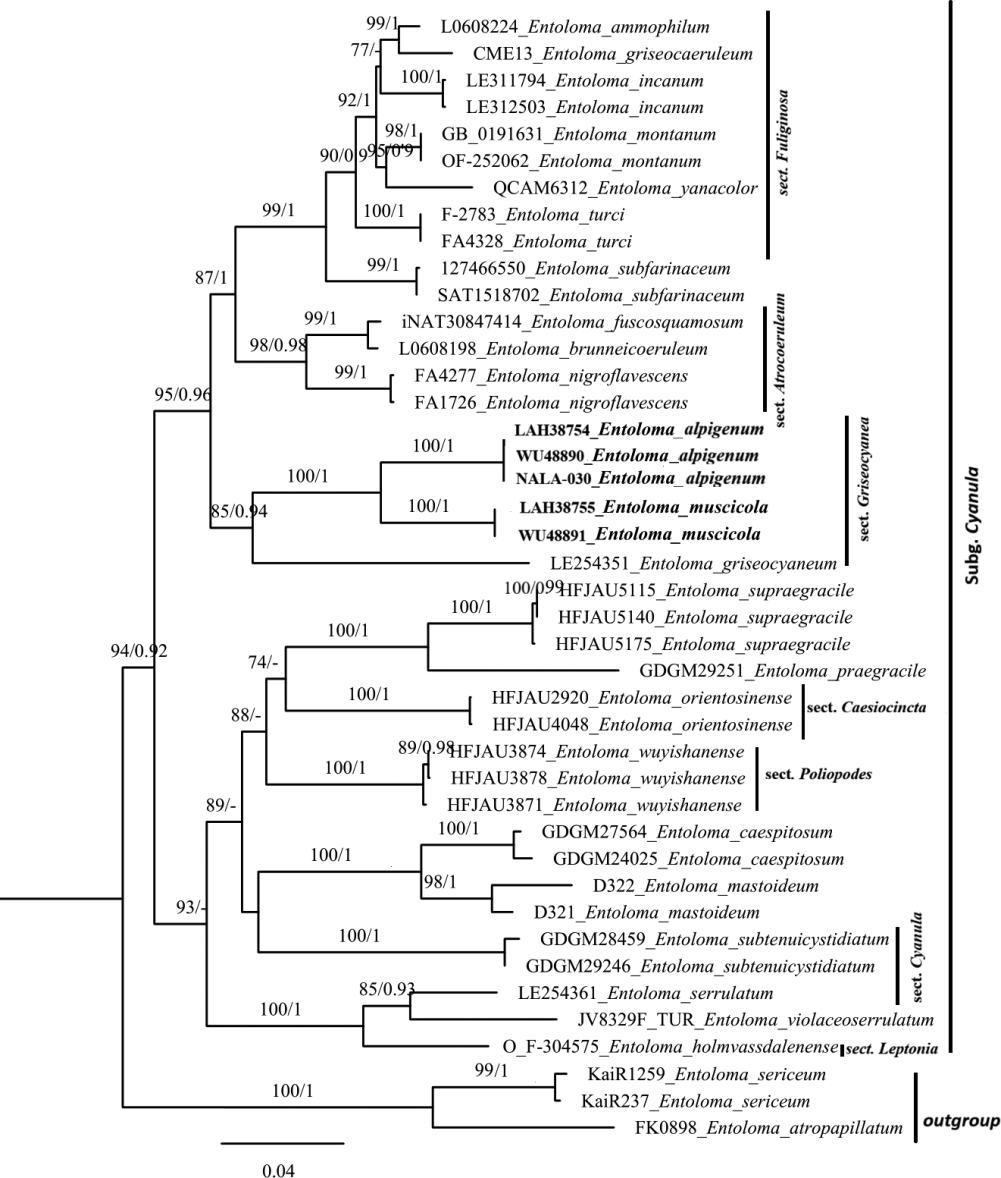
Phylogram of *Entoloma* subg. *Cyanula* spp. generated by Bayesian inference (BI) analysis based on dataset of nrITS, nrLSU mtSSU and *rpb*2 sequences. The tree is rooted with *E. sericeum* and *E. atropapillatum*. Bayesian inference (BI-PP) ≥ 0.90 and ML bootstrap proportions (ML-BP) ≥ 70% are indicated as PP/BP. The new taxa are marked in bold.

The phylogenetic tree is shown in Fig. 1. The two new species clustered within the subgenus *Cyanula* clade, forming a distinct and well-supported clade (100% BS/1.00 PP) as sister species.

### Taxonomy

#### Entoloma alpigenum

Taxon classificationFungiAgaricalesEntolomataceae

Khurshid & Naseer sp. nov.

15BE37E5-E21A-5C9B-BF4E-2B1116FDD1C2

861488

Figs 2, 3, 4

##### Etymology.

The specific epithet “alpigenum” is derived from “originating from alpine regions,” reflecting the species’ high-elevation distribution.

**Figure 2.**
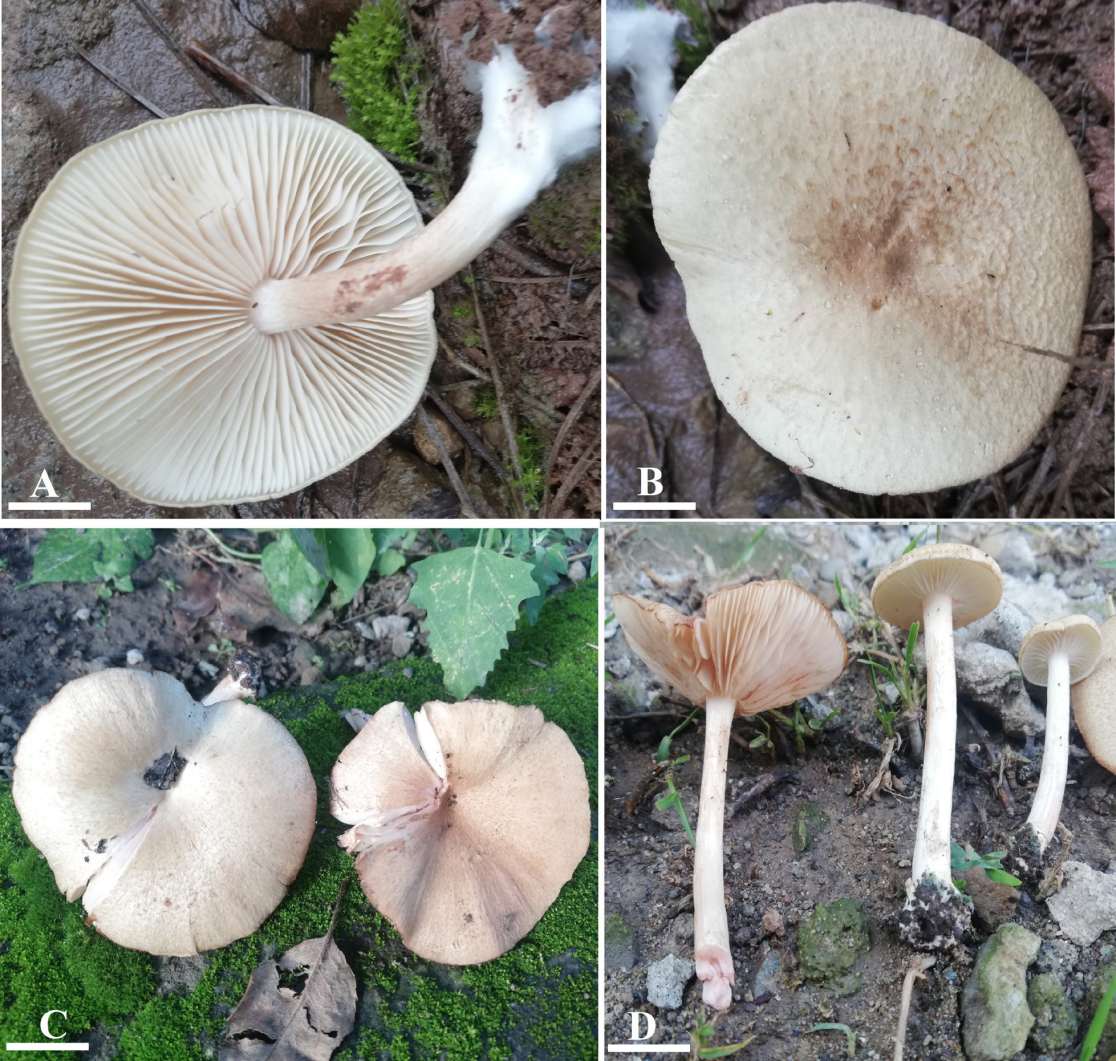
Morphology of *Entoloma alpigenum* (**A–D**) LAH38754, holotype. **A**. Lamellae and stipe view; **B, C**. Pileus view; **D**. Lamellae and stipe view. Scale bars: 1.7 cm (**A**); 1.5 cm (**B**); 4.1 cm (**C**); 3.3 cm (**D**).

**Figure 3.**
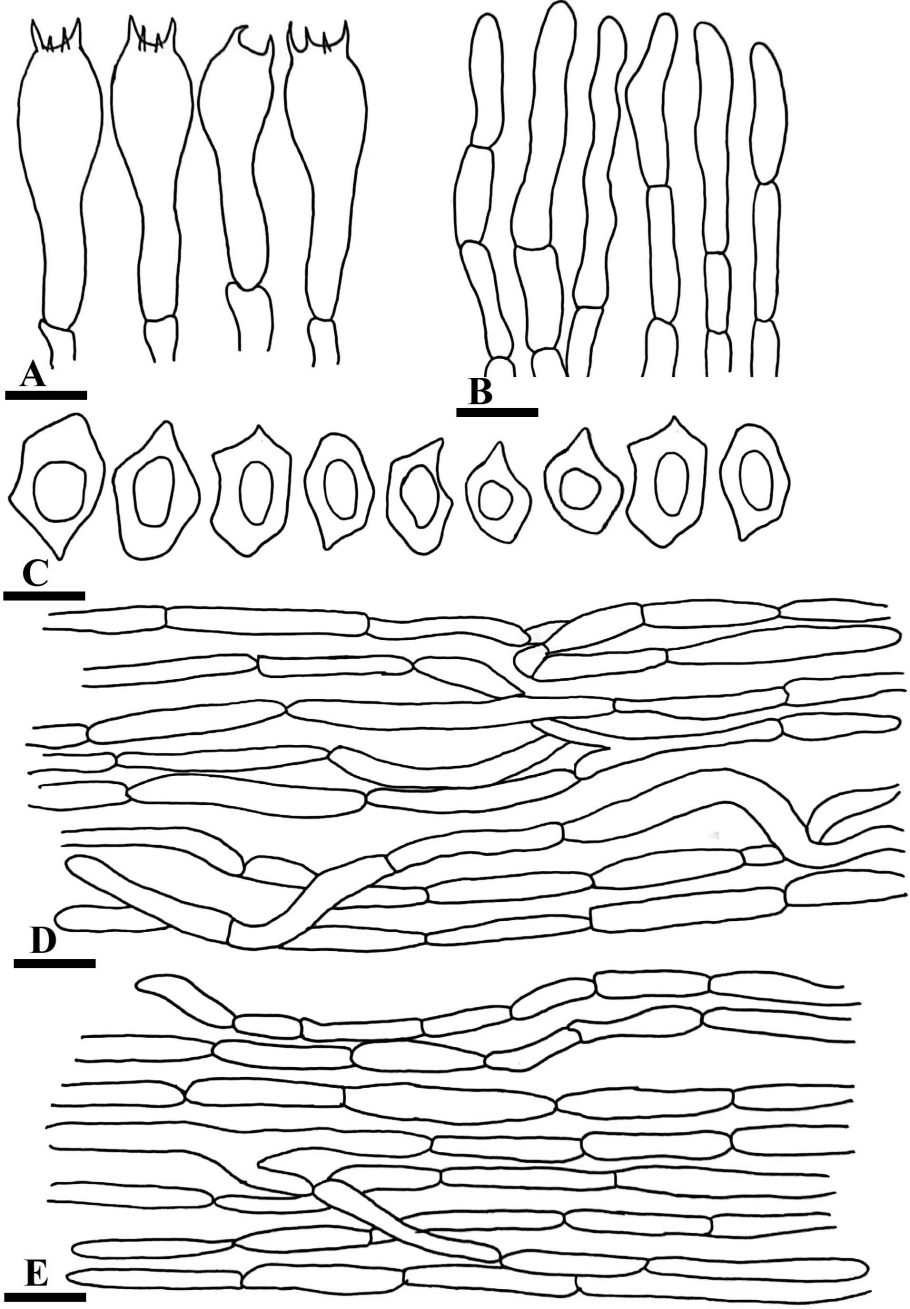
Anatomy of *Entoloma alpigenum* (**A–E**). **A**. Basidia; **B**. Cheilocystidia; **C**. Basidiospores; **D**. Piliepellis; **E**. Stipitipillis. Scale bars: 1.3 µm (**A**); 3 µm (**B**); 8 µm (**C**); 14 µm (**D**); 10 µm (**E**).

**Figure 4.**
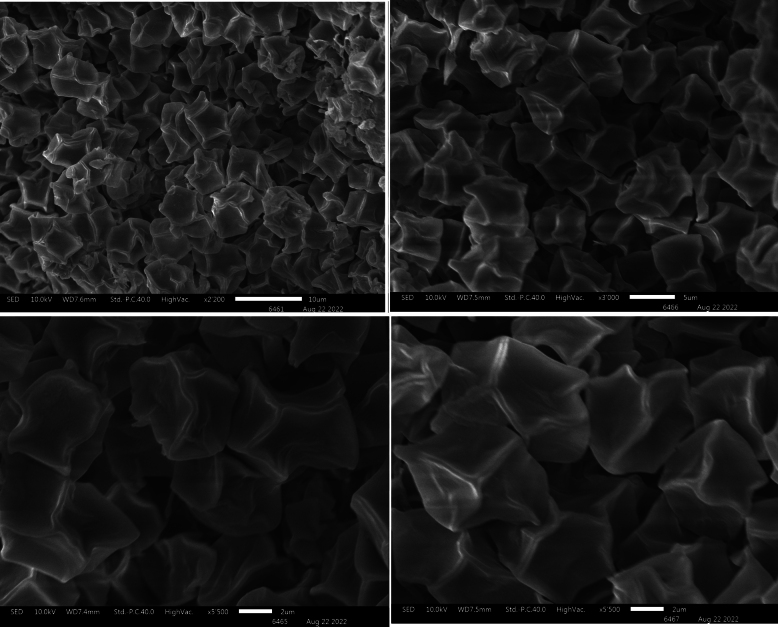
Scanning Electron Microscopy of *Entoloma alpigenum*.

##### Holotype.

Pakistan • Azad Jammu and Kashmir, Bagh District, Choki City, 33.98°N, 73.78°E, alt. 2,625 m, 20 September 2020, collected by Rubab Khurshid, LAH38754.

##### Diagnosis.

*Entoloma alpigenum* is characterized by its light brown, slightly squamulose pileus, whitish–cream context, lacking gray-blue pigments, cylindrical to flexuose cheilocystidia and heterodiametric, 5–7-angled basidiospores. In contrast, its closely related species *E. griseocyaneum* shows bluish tones and slightly different cheilocystidia. Its high-elevation alpine habitat (2,625 m) together with macroscopic and microscopic features clearly distinguish it from its close relatives.

##### Macromorphology.

Basidiomata medium-sized. Pileus 1.5–9.5 cm across, convex to plano-convex, becoming slightly depressed at the center with age. Surface dry, rugulose to finely squamulose at the disc, smoother toward the margin. Centre light brown (5YR 6/4), progressively becoming pale brown to cream (5Y 9/4) toward the margin. Margin entire to slightly striate, sometimes cracked in mature basidiomata. Context whitish to cream (5Y 9/2), unchanging when cut. Lamellae free, close, with entire to slightly serrulate edges, whitish to cream (10Y 9/2), becoming pale yellowish (10Y 9/4) in maturity. Lamellulae arranged in three to four tiers, irregular in length and distribution. Stipe 4.8–6.5 × 0.3–0.6 cm, cylindric, concolorous with the pileus surface; base whitish (5Y 9/2), becoming pale yellowish (5Y 9/4) toward the apex. Surface smooth; stipe stuffed; base subbulbous to slightly swollen, often attached to a whitish rhizomorphic mass. Annulus and volva absent. Smell and taste not recorded.

##### Micromorphology.

Basidiospores [60/3/2], (7.9–)9.1–10.8(–11.3) × (6.0–)6.2–8.8(–9.1) µm, av. L × av. W = 10.1 × 6.9 µm, Q = 1.1–1.6 µm, avQ = 1.39, heterodiametric, 5–7 angled in side view, with weak angles, smooth, thin-walled, epiculus present, non-granulate, mono gutulate, hyaline in 5% KOH. Basidia (38.3–)39.4–42.5(–49.2) × (7.1–)7.3–10.3(–11.2) µm, av. L × av. W = 40.6 × 8.9 µm, clavate, mostly 4-spored, occasionally 2-spored, with pointed sterigmata, non-guttulate, non-granulate, sessile at the base, and without clamp connections, hyaline in 5% KOH. Cheilocystidia (29.6–)31.2–36.7(–44.8) × (3.9–)4.1–5.4(–5.8) µm, av. L × av. W = 34.2 × 5.3 µm, cylindrical to flexuous, thin-walled, composed of 2–3 basal cells, without clamp connection, and hyaline in 5% KOH; pleurocystidia absent. Stipitipellis composed of septate, unbranched, thin-walled hyphae (10.1–)12.5–15.8(–17.4) µm in diameter, lacking clamp connections, with caulocystidia not observed. Pileipellis a cutis of crowded, filamentous, septate hyphae (2.8–)4.7–10.1(–11.4) µm in diameter, rarely branched, with well-differentiated terminal cells, clamp connections present, pileocystidia absent, non-guttulate, thin-walled, and hyaline in 5% KOH.

##### Habitat.

Solitary or scattered on soil or moss under coniferous forest.

##### Distribution.

Currently only known from Azad Jammu and Kashmir, Pakistan.

##### Additional specimen examined.

Pakistan • Azad Jammu and Kashmir, Bagh District, Ochar City, 33.98°N, 73.78°E, alt. 1,611 m, 24 August 2021, collected by Rubab Khurshid, WU48890; Pakistan • Azad Jammu and Kashmir, Bagh District, Ochar City, 33.98°N, 73.78°E, alt. 1,454 m, 28 August 2021, collected by Rubab Khurshid, Nala-030.

##### Notes.

*Entoloma alpigenum* belongs to the subgenus *Cyanula* and is most closely related to the European species *E. griseocyaneum*, but differs in macroscopic coloration, spore dimensions, and cystidial morphology. It is also comparable to Asian species such as *E. nigroflavescens* and *E. fuscosquamosum*. *Entoloma alpigenum* differs from *E. griseocyaneum* in its light brown, slightly squamulose pileus and pale yellowish lamellae, entirely lacking bluish pigmentation, whereas *E. griseocyaneum* is characterized by a grayish-blue to bluish-black pileus with bluish lamellae when young. In addition, *E. alpigenum* possesses smaller basidiospores (9.1–10.8), while *E. griseocyaneum* has comparatively larger spores (9–13.5) (Noordeloos 1992; Brandrud et al. 2012). Similarly, the pileus of *E. alpigenum* is paler and less distinctly fibrillose than the dark brown to blackish-brown, more strongly squamulose pileus of *E. nigroflavescens* and *E. fuscosquamosum* (Noordeloos 1992; Largent 1994a).

Phylogenetically, *Entoloma alpigenum* belong to the subgenus *Cyanula* as a distinct, well-supported lineage, forming a close relationship with *E. griseocyaneum*. Despite the close phylogenetic affinity, both species remain clearly separable by their morphological characters. Microscopically, *E. alpigenum* has larger basidiospores (9.1–10.8 × 6.2–8.8 µm) with 5–7 weak angles, which contrasts with the smaller, more sharply angular spores commonly found in *E. griseocyaneum* (Noordeloos 1992; Kokkonen 2015). The cylindrical to flexuose cheilocystidia of *E. alpigenum* (av. 39.6 × 5.3 µm) are also distinctive. In *E. griseocyaneum*, cheilocystidia when present are typically clavate to lageniform, not narrow and flexuose. In *E. fuscosquamosum*, cystidia are broader and fusiform, while *E. nigroflavescens* lacks cheilocystidia entirely (Noordeloos 1988; Noordeloos 1992; Buyck et al. 2022). The absence of pleurocystidia and predominantly four-spored basidia are shared with some related species, but the overall combination of characters is unique. *E. alpigenum* inhabits high-altitude coniferous forests (1,611–2,625 m a.s.l.) of the western Himalayas. In contrast, *E. griseocyaneum* is distributed mainly in temperate European moss-rich grasslands and woodland margins (Brandrud et al. 2012). Both *E. fuscosquamosum* and *E. nigroflavescens* generally occur at lower elevations and in more temperate forested habitats (Noordeloos 1992). *E. alpigenum* is strongly supported by three collections, confirming its distinct species identity.

#### Entoloma muscicola

Taxon classificationFungiAgaricalesEntolomataceae

Khurshid & Naseer sp. nov.

1F02FE55-4D42-5DD5-98E3-862934A7F782

861489

Figs 5, 6, 7

##### Etymology.

The specific epithet “muscicola” refers to the moss-dwelling habit of the species.

**Figure 5.**
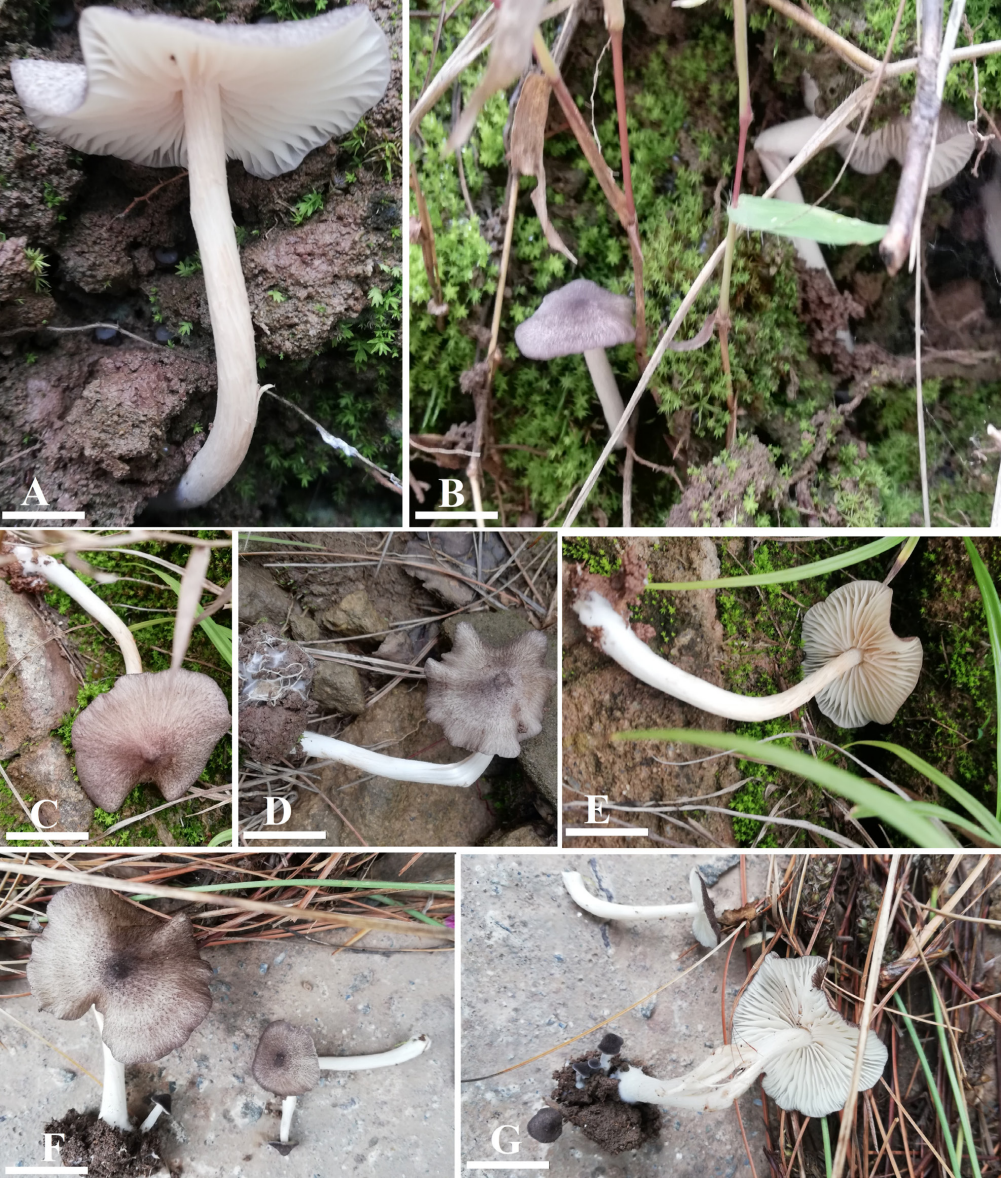
Morphology of *Entoloma muscicola* (**A–G**) LAH38755, holotype. **A**. Lamellae and stipe view; **B–D**. Pileus and stipe view; **E**. Lamellae and stipe view; **F**. Pileus and stipe view; **G**. Lamellae and stipe view. Scale bars: 1 cm (**A**); 3.5 cm (**B**); 2.5 cm (**C**); 2 cm (**D–F**).

**Figure 6.**
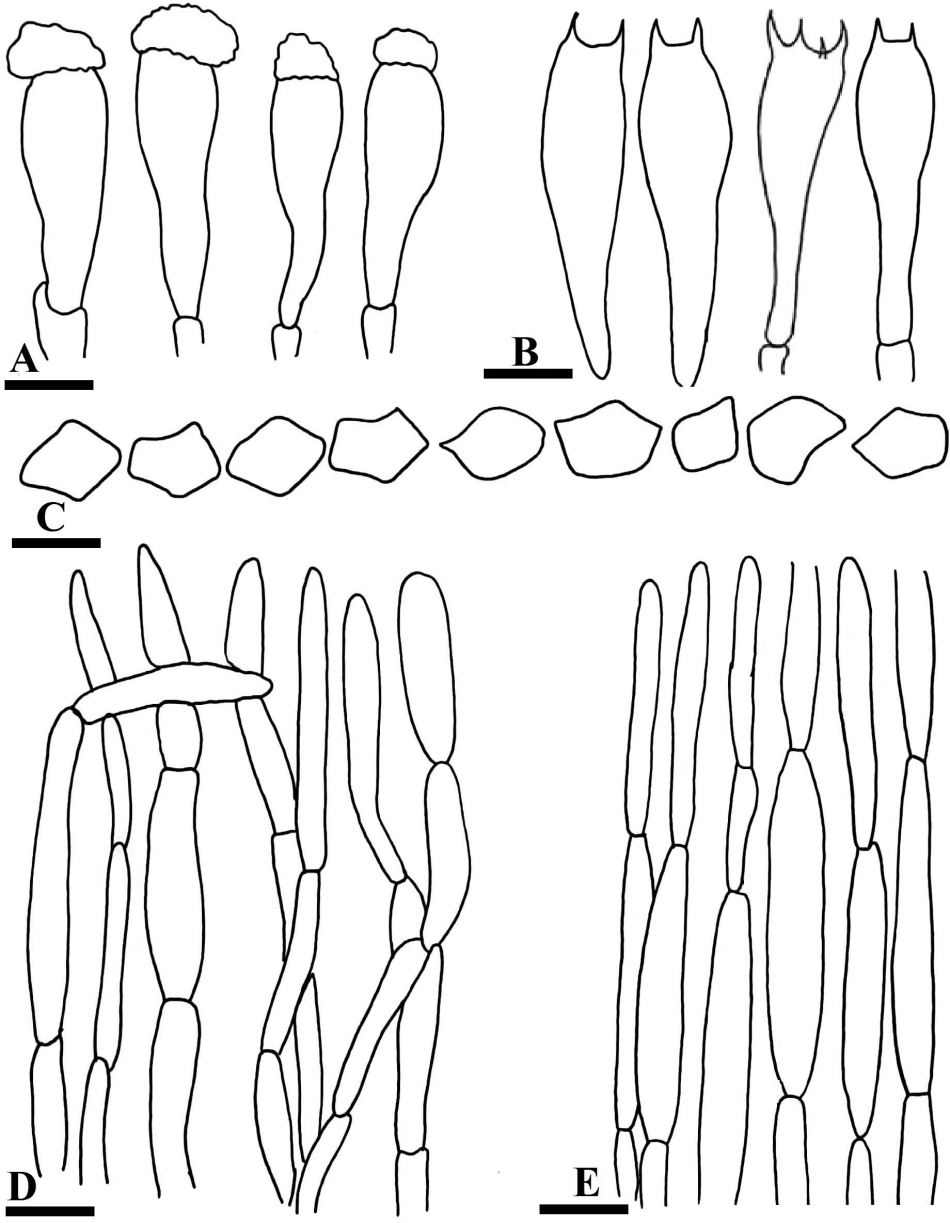
Anatomy of *Entoloma muscicola* (**A–E**). **A**. Cheilocystidia; **B**. Basidia; **C**. Basidiospores; **D**. Pileipellis; **E**. Stipitipellis. Scale bars: 8 µm (**A**); 10 µm (**B**); 7 µm (**C**); 12 µm (**D**); 10 µm (**E**).

**Figure 7.**
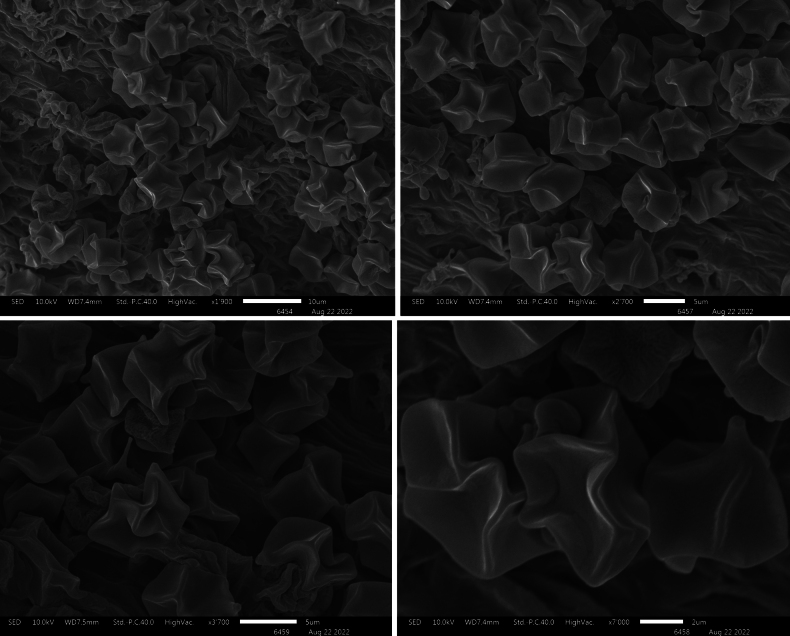
Scanning Electron Microscopy of *Entoloma muscicola*.

##### Holotype.

Pakistan • Azad Jammu and Kashmir, Bagh District, Choki City, 33.98°N, 73.78°E, alt. 1,811 m, 24 August 2020, collected by Rubab Khurshid (LAH38755).

##### Diagnosis.

*Entoloma muscicola* is characterized by cuspidate pileus, distant lamellae, weakly angled mono-gutulate spores, distinctive clavate cheilocystidia and the absence of cellular pigments and clamp connections in the hyphae distinguishing it from closely related species including *E. griseocyaneum*, *E. fuscosquamosum*, and *E. nigroflavescens*.

##### Macromorphology.

Basidiomata small to medium-sized. Pileus 1.0–5.7 cm across, ovoid to conical when young, becoming campanulate to cuspidate at maturity, with a distinctly darker deep brown umbonate center (2.5YR 1/8) gradually fading to light brown toward the margin (7.5YR 5/4). Surface dry, smooth to subtly shiny, striate to plicate toward the margin; margin wavy, striate, sometimes slightly uplifted. Context whitish to cream (5Y 9/2), unchanged when cut. Lamellae free, distant, with entire and even edges, whitish (10Y 9/2) to pale yellow (10Y 9/4). Lamellulae present, arranged in 3–4 tiers, regularly distributed. Stipe 3–7.4 × 0.2–0.5 cm, cylindrical, whitish (5Y 9/2) at the base becoming pale yellowish (5Y 9/4) toward the apex, surface smooth, hollow, subbulbous to slightly swollen at the base, often attached to a mass of whitish rhizomorphs. Annulus absent. Volva absent. Odor and taste not recorded.

##### Micromorphology.

Basidiospores [60/3/2], (9.3–)9.7–11.6(–12.5) × (6.1–)6.3–7.9(–8.2) µm, av. L × av. W = 10.8 × 6.9 µm, Q = 1.2(–1.34)–1.7, avQ = 1.6, heterodiametric, 4–6 angled in side view, with weak angles; smooth, thin-walled; epiculus present; non-granulate; mono-guttulate; hyaline in 5% KOH. Basidia (34.4–)36.4–43.8(–46.0) × (8.8–)9.4–10.1(–10.4) µm, av. L × av. W = 39.3 × 10.0 µm clavate, tapering sharply toward the base; usually two sterigmata, rarely four; pointed ends; non-guttulate, non-granulated; base sessile; clamp connection absent; hyaline in 5% KOH. Cheilocystidia (30–)34.1–39.8(–45) × (7.8–)8.6–9.4(–10.5) µm, av. L × av. W = 36.8 × 9.1 µm, clavate, apex covered with wavy cap structure, thin walled, 1 celled base, hyaline in 5% KOH. Stipitipellis a cutis, hyphae (5.1–)5.6–6.8(–12.3) µm in diameter, septate, unbranched, thin walled, clamp connections absent, caulocystidia not observed, hyaline in 5% KOH. Pileipellis a cutis, hyphae (8.6–)8.8–12.5(–14.2) µm in diameter, septate, filamentous, crowded, un-branched, with well differentiated terminal cells, clamp connection absent, pileocystidia absent, non-guttulate, thin walled, hyaline in 5% KOH.

##### Habitat.

Found solitary or gregarious, dwelling on soil or moss under conifers.

##### Distribution.

Currently only known from Azad Jammu and Kashmir, Pakistan

##### Additional material examined.

Pakistan • Azad Jammu and Kashmir, Bagh District, Choki City, 33.98°N, 73.78°E, alt. 1,521 m, 28 August 2021, Collected by Rubab Khurshid, WU48891.

##### Notes.

*Entoloma muscicola* is closely related to *E. griseocyaneum*, *E. nigroflavescens*, and *E. fuscosquamosum* but can be readily distinguished by a combination of morphological and ecological traits. Compared with *E. griseocyaneum*, *E. muscicola* is readily distinguished by lacking gray-blue pigmentation in all parts of the basidiome and by having a brown to deep brown, cuspidate pileus with whitish to pale yellow lamellae. In contrast, *E. griseocyaneum* is characterized by bluish to gray-blue tones on the pileus and stipe and by producing distinctly more slender basidiomes (Fries 1821; Noordeloos 1992). *Entoloma muscicola* has clavate cheilocystidia with a wavy apical cap, and weakly 4–6-angled mono-gutulate basidiospores (av. 10.8 × 6.9 µm). *E. griseocyaneum* lacks cheilocystidia on the lamellar edge and produces 6-angled basidiospores (Noordeloos 1992; Buyck et al. 2022). Basidia in *E. muscicola* are predominantly 2-spored, rarely 4-spored, whereas related taxa *E. fuscosquamosum* and *E. griseocyaneum* typically have 4-spored basidia (Noordeloos 1992; Largent 1994a). Cheilocystidia in *E. muscicola* (36.8 × 9.1 µm) distinguish it from *E. nigroflavescens*, which lacks cheilocystidia, and from *E. fuscosquamosum*, which possesses broader, fusiform cheilocystidia (Noordeloos 1992; Buyck et al. 2022). The pileipellis forms a cutis of crowded filamentous hyphae with well-differentiated terminal cells, and clamp connections are absent. *Entoloma muscicola* occurs on soil or moss under montane mixed forests (*Pinus wallichiana* and *Cedrus deodara*) at ~1,811 m a.s.l., whereas *E. griseocyaneum* is typically associated with grasslands or subalpine pastures at lower to mid-elevations (Buyck et al. 2022) and *E. fuscosquamosum* occurs in temperate forests (Largent 1994b). Based on these differences and phylogeny, we introduce *E. muscicola* in section *Griseocyanea*; subgenus *Cyanula*, as a distinct species.

## Discussion

The present study significantly expands the documented diversity of *Entoloma* subgenus *Cyanula* in Pakistan and underscores the systematic complexity of this lineage in montane ecosystems. The recognition of *E. muscicola* and *E. alpigenum* introduces two previously undocumented phylogenetic lineages, indicating that *Cyanula* remains insufficiently explored in South Asia despite its global taxonomic importance within the genus. Both species are robustly assigned to *Cyanula* based on concordant evidence from diagnostic morphological characters and multilocus phylogenetic analyses. Phylogenetic reconstruction resolves *E. muscicola* and *E. alpigenum* as strongly supported sister taxa that are clearly distinct from morphologically similar species such as *E. nigroflavescens* and *E. fuscosquamosum*, supporting their interpretation as independent evolutionary lineages rather than geographic variants of previously described taxa. The close correspondence between micromorphological traits, particularly basidiospore morphology and cheilocystidial structure and molecular divergence, further substantiates their recognition as discrete species and contributes to refining species boundaries within *Entoloma*.

The occurrence of both taxa in high-altitude mixed forests suggests that elevational gradients may play an important role in diversification processes within *Entoloma*. *Entoloma alpigenum*, recorded at 2,625 m a.s.l., inhabits cooler, more humid, and comparatively undisturbed environments than many related lowland species. Such ecological conditions may facilitate isolation, limit gene flow, and promote adaptive differentiation, thereby fostering speciation in montane lineages of *Cyanula*. Although broader sampling is needed to rigorously test this hypothesis, the present findings indicate that high-elevation habitats in Pakistan likely represent significant reservoirs of undescribed fungal diversity.

Overall, this study highlights the value of integrative taxonomic approaches for revealing cryptic diversity in *Entoloma* and identifies high-altitude ecosystems in Pakistan as important yet underexplored centers of fungal biodiversity. Beyond its systematic implications, documenting diversity within *Entoloma* also holds applied relevance, as species of the genus are known to produce bioactive metabolites with potential agricultural, medicinal, and industrial applications.

## Supplementary Material

XML Treatment for Entoloma alpigenum

XML Treatment for Entoloma muscicola

## Additional information

### Use of AI

No use of AI was reported.

### Author ORCIDs

Rubab Khurshid https://orcid.org/0000-0001-6542-817X

Arooj Naseer https://orcid.org/0000-0002-4458-9043

Qirui Li https://orcid.org/0000-0001-8735-2890

Abdul Nasir Khalid https://orcid.org/0000-0002-5635-8031

### Data availability

All of the data that support the findings of this study are available in the main text.
